# Predictive markers of response to immune checkpoint inhibitor rechallenge in metastatic non-small cell lung cancer

**DOI:** 10.37349/etat.2024.00275

**Published:** 2024-10-18

**Authors:** Aram A. Musaelyan, Svetlana V. Odintsova, Karina A. Musaelyan, Magaripa A. Urtenova, Ekaterina P. Solovyova, Lyubov I. Menshikova, Sergey V. Orlov

**Affiliations:** IRCCS Istituto Romagnolo per lo Studio dei Tumori (IRST) “Dino Amadori”, Italy; ^1^Department of Clinical Oncology, Pavlov First Saint Petersburg State Medical University, 197022 Saint Petersburg, Russia; ^2^EuroCityClinic LLC, 197022 Saint Petersburg, Russia; ^3^Almazov National Medical Research Centre, 197341 Saint Petersburg, Russia; ^4^Department of Clinical Oncology, Arkhangelsk Clinical Oncology Center, 163045 Arkhangelsk, Russia; ^5^I.V. Kurchatov Complex for Medical Primatology, National Research Centre “Kurchatov Institute”, 354376 Sochi, Russia

**Keywords:** Immune checkpoint inhibitors, non-small cell lung cancer, rechallenge, objective response, ECOG, neutrophil-to-lymphocyte ratio, predictive marker

## Abstract

**Aim:**

The present study aims to evaluate the efficacy of rechallenge with immune checkpoint inhibitors (ICIs) compared to chemotherapy and the predictive role of clinical parameters in non-small cell lung cancer (NSCLC) patients who were rechallenged.

**Methods:**

The study included 113 metastatic NSCLC patients who had initially responded to ICIs and platinum-based chemotherapy, either in combination in the first line or sequentially in the first and second line, but later experienced disease progression. Of those patients, 52 later received ICI rechallenge and 61 were exposed to chemotherapy.

**Results:**

In the rechallenge cohort, the median age was 67 years, 38 patients were men (73.1%), 26 (50.0%) had squamous cell carcinoma. Patients who underwent ICI rechallenge had longer overall survival (OS) compared to those who received chemotherapy (12.9 months vs. 9.6 months, *P* = 0.008). Multivariate analysis for progression-free survival (PFS) and OS revealed that poor Eastern Cooperative Oncology Group Performance Status (ECOG PS; PFS: *P* = 0.013 and OS: *P* = 0.037), absence of objective response during initial ICI therapy (PFS: *P* = 0.014 and OS: *P* = 0.028), and baseline neutrophil-to-lymphocyte ratio (NLR) ≥ 3.8 (PFS: *P* = 0.001 and OS: *P* = 0.003) were negative predictive factors of ICI rechallenge. The three parameters were included in a risk model named as the NEO score, which stratified patients who received ICI rechallenge into two predictive groups. Patients with ECOG PS 0-1, objective response during initial ICI treatment, and NLR < 3.8 (favorable group) had longer PFS (8.6 months vs. 3.0 months, *P* < 0.001) and OS (16.6 months vs. 5.5 months, *P* < 0.001) compared to those with absence of all three markers (poor group). There was no association between the NEO score and survival outcomes in patients who did not undergo rechallenge.

**Conclusions:**

ICI rechallenge showed a survival benefit, particularly in NSCLC patients with NLR < 3.8, good ECOG PS, and objective response.

## Introduction

The use of immune checkpoint inhibitors (ICIs) is the mainstay of treatment for patients with metastatic non-small cell lung cancer (NSCLC) without targetable driver mutations [1]. ICIs, such as anti-programmed cell death protein 1 (PD-1)/programmed cell death-ligand 1 (PD-L1) antibodies, were initially approved as second-line monotherapy in metastatic NSCLC, showing a survival benefit compared to docetaxel monotherapy [2, 3]. Anti-PD-1/PD-L1 antibodies have been later approved for use in the first-line setting as monotherapy or in combination with chemotherapy and/or other ICIs, such as anti-CTLA-4 antibodies, depending on the level of PD-L1 expression [1, 3]. Recently, ICIs are increasingly being used in the neoadjuvant and adjuvant settings for patients with resectable NSCLC [1]. Several NSCLC patients who receive ICIs experience a long-term response to the therapy [1]. However, ICIs ultimately need to be discontinued due to disease progression, severe immune-related adverse events (irAEs), or reaching a predetermined number of treatment cycles [4].

Therapeutic options for metastatic NSCLC patients without targetable driver genetic alterations who have experienced disease progression after ICIs and platinum-based chemotherapy are limited [5]. The main subsequent therapy approach is chemotherapy alone or in combination with angiogenesis inhibitors [5]. However, these therapies in advanced NSCLC have shown limited efficacy, with a median overall survival (OS) of 7.3 months [6]. Considering the dynamic intratumoral heterogeneity, rechallenge with ICIs may be a promising option, especially for patients who have previously responded to the therapy [4, 7, 8]. A retrospective analysis of a phase III trial revealed that patients who continued to receive atezolizumab treatment after disease progression had a notably longer median OS [9]. A recent meta-analysis, which primarily included small retrospective cohort studies, also demonstrated promising long-term efficacy of ICI rechallenge in NSCLC patients [10]. However, the retreatment strategy is effective for only a limited number of patients [11]. Therefore, it is essential to identify patients who will benefit the most from ICI rechallenge in order to prevent undesired adverse events and reduce the financial strain caused by the indiscriminate use of expensive therapy [12].

PD-L1 expression in tumor is currently the only approved predictive marker in clinical practice for initial ICI therapy in metastatic NSCLC [13]. However, several studies have failed to conclusively show the predictive value of PD-L1 expression in ICI rechallenge [11, 14, 15]. The antitumor response to ICI rechallenge is also largely dependent on the tumor microenvironment [16]. Inflammatory indices, such as the neutrophil-to-lymphocyte ratio (NLR), provide a non-invasive reflection of the immune background in the microenvironment [17, 18]. NLR may serve as a predictor of survival outcomes on ICI rechallenge [15]. Another host’s parameter that could differentiate responders from non-responders to ICI rechallenge is the general condition, evaluated using the Eastern Cooperative Oncology Group Performance Status (ECOG PS) scale [15]. However, single markers do not accurately represent tumor-host interactions during ICI rechallenge. Combining these markers could address this limitation. In the present study, we evaluated the efficacy of ICI rechallenge compared to standard therapy and predictive role of clinical parameters in rechallenged NSCLC patients.

## Materials and methods

### Patients

**Figure 1:**
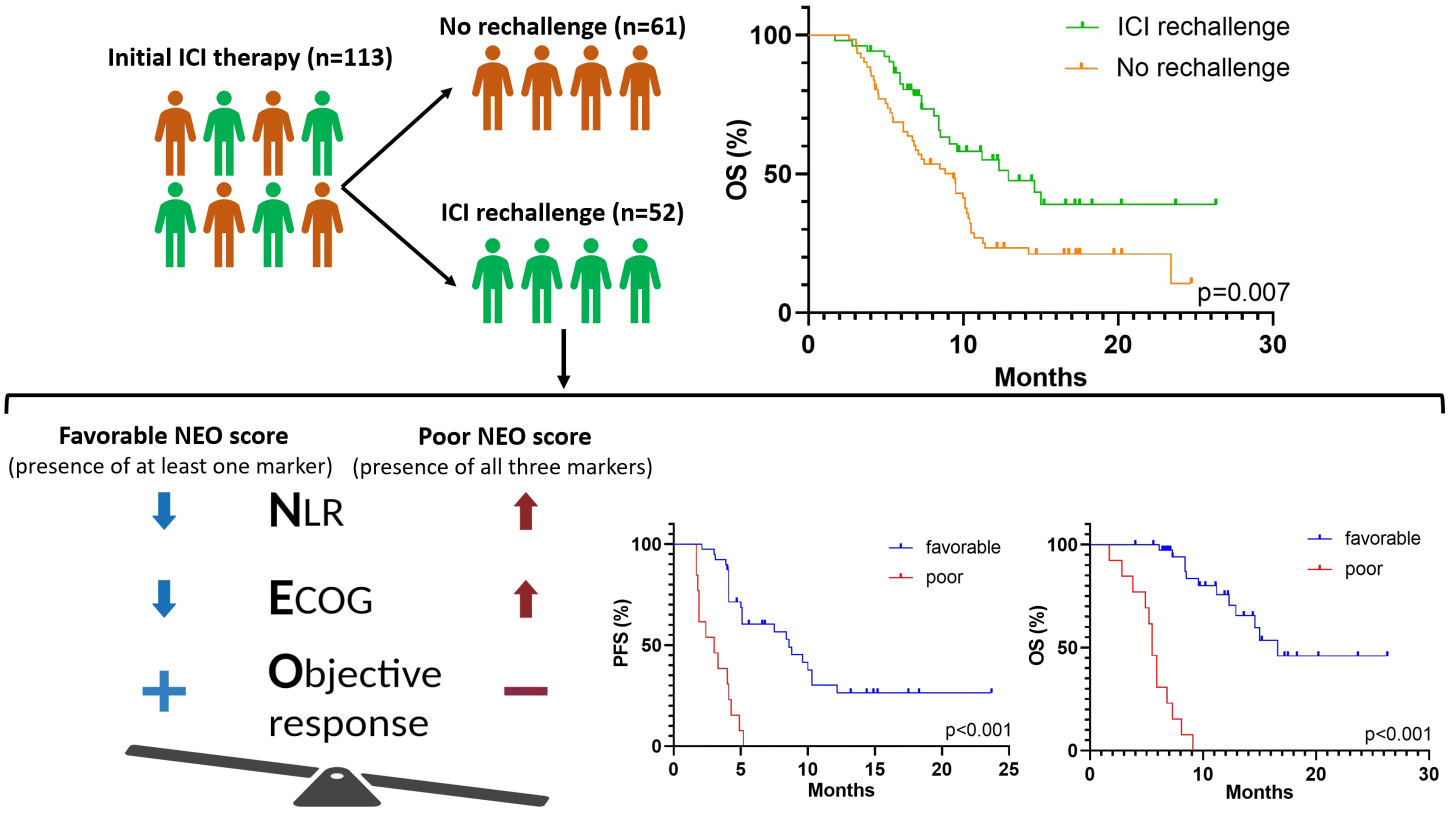
Graphical abstract displaying the survival benefit of ICI rechallenge in ICI-pretreated NSCLC, and predictive markers of retreatment efficacy: NLR < 3.8, ECOG PS 0-1, and objective response. The NEO score, using these markers, accurately predicts PFS and OS. ECOG PS: Eastern Cooperative Oncology Group Performance Status; NLR: neutrophil-to-lymphocyte ratio; OS: overall survival; PFS: progression-free survival; ICI: immune checkpoint inhibitor; NSCLC: non-small cell lung cancer

The retrospective study included 113 metastatic NSCLC patients without targetable alterations who responded to initial ICIs for at least 4 months. Patients initially received first-line ICIs in combination with platinum-based chemotherapy or were treated sequentially with these drugs in the first and second line. Subsequently, patients discontinued ICI-containing treatment due to disease progression. Of these patients, 52 were retreated with ICIs (rechallenge cohort) and 61 later underwent chemotherapy with or without antiangiogenic agents (no rechallenge cohort). The “no rechallenge cohort” was collected to compare the efficacy of ICI rechallenge and evaluate the predictive value of markers. The workflow of this study is provided in Figure 1.

All patients were treated according to standard clinical practice between March 2018 and April 2024 at the Pavlov First Saint Petersburg State Medical University, at the EuroCityClinic LLC (both at Saint Petersburg, Russia) and at the Arkhangelsk Clinical Oncology Center (Arkhangelsk, Russia). The patients included in the study were in compliance with the Helsinki Declaration and were approved by the ethics committee of Pavlov First Saint Petersburg State Medical University (approval no. 119-2024). Written informed consent was obtained from all participants in the study.

The inclusion criteria for both cohorts were as follows: (1) age ≥ 18 years; (2) histologically confirmed NSCLC; (3) metastatic disease [stage IV according to the tumor node metastasis staging and the American Joint Committee on Cancer (8th edition, 2017)] [19]; (4) ECOG PS 0-2; (5) absence of targetable alterations (EGFR mutations, ALK and ROS1 translocations) in the case of non-squamous cell carcinoma; (6) at least 4 months on initial ICI therapy; (7) disease progression on the initial ICI therapy; (8) at least 3 cycles of subsequent therapy (ICI rechallenge or chemotherapy). The exclusion criteria were as follows: (1) previous or ongoing autoimmune disease; (2) the presence of concurrent other malignancies; (3) discontinuation of initial ICI therapy due to immune-related toxicity.

### Data collection

The clinical data of the patients was collected retrospectively from their medical records, including sex, age, body mass index (BMI), smoking status, histological type, PD-L1 immunohistochemistry (IHC) expression at initial treatment, molecular status, number of metastases and their sites at the start of subsequent line therapy (ICI rechallenge or chemotherapy), ECOG PS and NLR at the start of subsequent line therapy, and treatment information.

The tumor’s PD-L1 expression was assessed as part of the standard procedure prior to the start of initial ICI therapy using IHC kits, including Dako PD-L1 clone 22C3 pharmDx (Agilent Technologies, Inc.) and Ventana PD-L1 clone SP142 (Ventana Medical Systems, Inc.). Data on NLR were collected from routine blood tests at the baseline of subsequent line therapy in both cohorts.

The Immune Response Evaluation Criteria in Solid Tumors (iRECIST) were used to analyze the tumor response to ICIs, while RECIST 1.1 was used to evaluate the response to chemotherapy [20, 21]. The best overall response was assessed according to iRECIST criteria, which includes complete and partial response (PR, both defined as objective response), stable disease (SD), and progressive disease (PD). irAEs during initial therapy and ICI rechallenge were also collected. The grading of irAEs was done according to the criteria of the National Cancer Institute Common Terminology Criteria for Adverse Events version 5.0 [22].

### Statistical analysis

The study endpoints were objective response rate (ORR), progression-free survival (PFS) and OS. ORR represents the percentage of patients who achieved partial and complete responses (CRs). PFS is defined as the time in months from the start of subsequent line treatment (ICI therapy in the rechallenge cohort and chemotherapy in the no rechallenge cohort) until disease progression, the date of the last patient contact, or follow-up date. OS in both cohorts is the period from the start of subsequent line therapy to death, the date of the last patient contact, or follow-up date.

The chi-squared test was used to compare qualitative variables. Receiver operating characteristic analysis was utilized to determine the optimal cut-off values for PD-L1 expression level and NLR. Survival curves for PFS and OS were generated using the Kaplan-Meier method, and differences were compared using the log-rank test. The Cox proportional hazards model was utilized in both univariate and multivariate analyses to determine the relationship between survival outcomes and the potential predictive parameters. The statistical analyses were performed using GraphPad Prism (v.9.5.0; GraphPad Software, Inc.).

## Results

### Patient characteristics

A total of 113 patients who initially received ICI therapy were included in the study: 52—in the rechallenge cohort and 61—in the no rechallenge cohort. In the rechallenge cohort, the median age was 67 years (range 39–86 years), with a majority of male patients (*n* = 38, 73.1%) and a predominance of a smoking history (*n* = 33, 63.5%), 26 (50%) had squamous cell carcinoma. In the rechallenge cohort, 37 patients (71.2%) underwent ICI combination therapy (platinum-containing therapy with or without angiogenesis inhibitor) in first-line settings and 15 patients (28.8%) initially received ICI monotherapy and platinum-based chemotherapy sequentially in the first and second line. Among patients in the rechallenge cohort, 29 patients (55.8%) additionally received chemotherapy after initial therapy with ICIs and platinum-based drugs, and 37 patients (71.2%) at the time of retreatment switched to a different ICI drug.

In the no rechallenge cohort, the median age was 65 years, ranging from 35 years to 85 years. The majority of patients were male (*n* = 44, 72.9%), 37 (60.7%) had a history of smoking, 33 (54.1%) had squamous cell carcinoma. In the cohort without ICI retreatment, 15 patients (24.6%) initially received sequentially ICI monotherapy and platinum chemotherapy, while 46 patients (75.4%) were treated with ICI combination therapy (platinum-containing therapy with or without angiogenesis inhibitor). The demographics, clinical, pathological and treatment data of the patients in both cohorts are shown in Table 1.

**Table 1:** The primary baseline characteristics of patients in rechallenge and no rechallenge cohorts

**Characteristics**	**Rechallenge cohort** **(*n* = 52)**	**No rechallenge cohort** **(*n* = 61)**
Sex, *n* (%)		
Male	38 (73.1)	44 (72.1)
Female	14 (26.9)	17 (27.9)
Age, median (IQR)	67 (57–72)	65 (55–71)
Age, *n* (%)		
< 65	23 (44.2)	29 (47.5)
≥ 65	29 (55.8)	32 (52.5)
ECOG PS at subsequent line		
0-1	37 (71.2)	47 (77.0)
2	15 (28.8)	14 (23.0)
Smoking status		
Ever smoking	34 (65.4)	37 (60.7)
Never smoking	18 (34.6)	24 (39.3)
Histological type, *n* (%)		
Adenocarcinoma	24 (46.2)	25 (41.0)
Squamous cell carcinoma	26 (50.0)	33 (54.1)
Other NSCLC	2 (3.8)	3 (4.9)
PD-L1 IHC expression		
< 1%	16 (30.7)	20 (32.8)
≥ 1 and < 49%	22 (42.3)	30 (49.2)
≥ 50%	14 (26.9)	11 (18.0)
Brain metastasis at ICI rechallenge		
Yes	12 (23.1)	17 (27.9)
No	40 (76.9)	44 (72.1)
Line of initial ICI therapy, *n* (%)		
1	44 (84.6)	55 (90.2)
2	8 (15.4)	6 (9.8)
Initial ICI regimen, *n* (%)		
Pembrolizumab + paclitaxel + carboplatin	19 (36.6)	25 (41.0)
Pembrolizumab + pemetrexed + cis-/carboplatin	12 (23.1)	13 (21.3)
Atezolizumab + paclitaxel + carboplatin + bevacizumab	6 (11.5)	8 (13.1)
Atezolizumab	6 (11.5)	4 (6.6)
Pembrolizumab	5 (9.6)	6 (9.8)
Nivolumab	4 (7.7)	5 (8.2)
Best overall response to initial ICI therapy, *n* (%)		
Complete response	1 (1.9)	0 (0.0)
PR	22 (42.3)	26 (42.6)
SD	25 (48.1)	30 (49.2)
Progressive disease	4 (7.7)	5 (8.2)
Any irAEs at initial ICI therapy, *n* (%)	23 (44.2)	25 (41.0)
Thyroid dysfunction	9 (17.3)	10 (16.4)
Skin reactions	6 (11.5)	5 (8.2)
Hepatitis	5 (9.6)	7 (11.5)
Pneumonitis	3 (5.8)	3 (4.9)
Chemotherapy regimen at subsequent line	0 (0.0)	
Docetaxel monotherapy		29 (47.5)
Docetaxel + angiogenesis inhibitor (bevacizumab/nintedanib/ramucirumab)		16 (26.2)
Pemetrexed		7 (11.5)
Afatinib		5 (8.2)
Gemcitabine		4 (6.6)
Treatment between two lines of ICI		0 (0.0)
Chemotherapy ± angiogenesis inhibitor	29 (55.8)	
No	23 (44.2)	
ICI rechallenge regimen		0 (0.0)
Pembrolizumab	20 (38.4)	
Nivolumab	17 (32.7)	
Atezolizumab	7 (13.5)	
Pembrolizumab + paclitaxel + carboplatin	4 (7.7)	
Pembrolizumab + pemetrexed + carboplatin	4 (7.7)	
Rechallenge with the same ICI		0 (0.0)
Yes	15 (28.8)	
No	37 (71.2)	
Any irAEs at ICI rechallenge, *n* (%)	18 (34.6)	0 (0.0)
Thyroid dysfunction	6 (11.5)	
Hepatitis	6 (11.5)	
Skin reactions	3 (5.8)	
Pneumonitis	3 (5.8)	

IQR: interquartile range; ECOG PS: Eastern Cooperative Oncology Group Performance Status; IHC: immunohistochemistry; ICI: immune checkpoint inhibitor; irAEs: immune-related adverse events; NSCLC: non-small cell lung cancer; PD-L1: programmed cell death-ligand 1; PR: partial response; SD: stable disease

### Efficacy of ICI rechallenge

The ORR in patients receiving ICI rechallenge was 3/52 (5.8%). All of these patients experienced a PR. No statistically significant association was observed between ORR and characteristics, such as sex, age, BMI, smoking status, histological type, PD-L1 IHC expression, number, and location of metastases, ECOG PS, NLR at the start of ICI rechallenge, line of initial therapy and its regimen, duration of initial ICI therapy, occurrence of irAEs during initial and subsequent therapy, ICI rechallenge regimen, and line of ICI rechallenge (*P* > 0.05).

**Figure 2:**
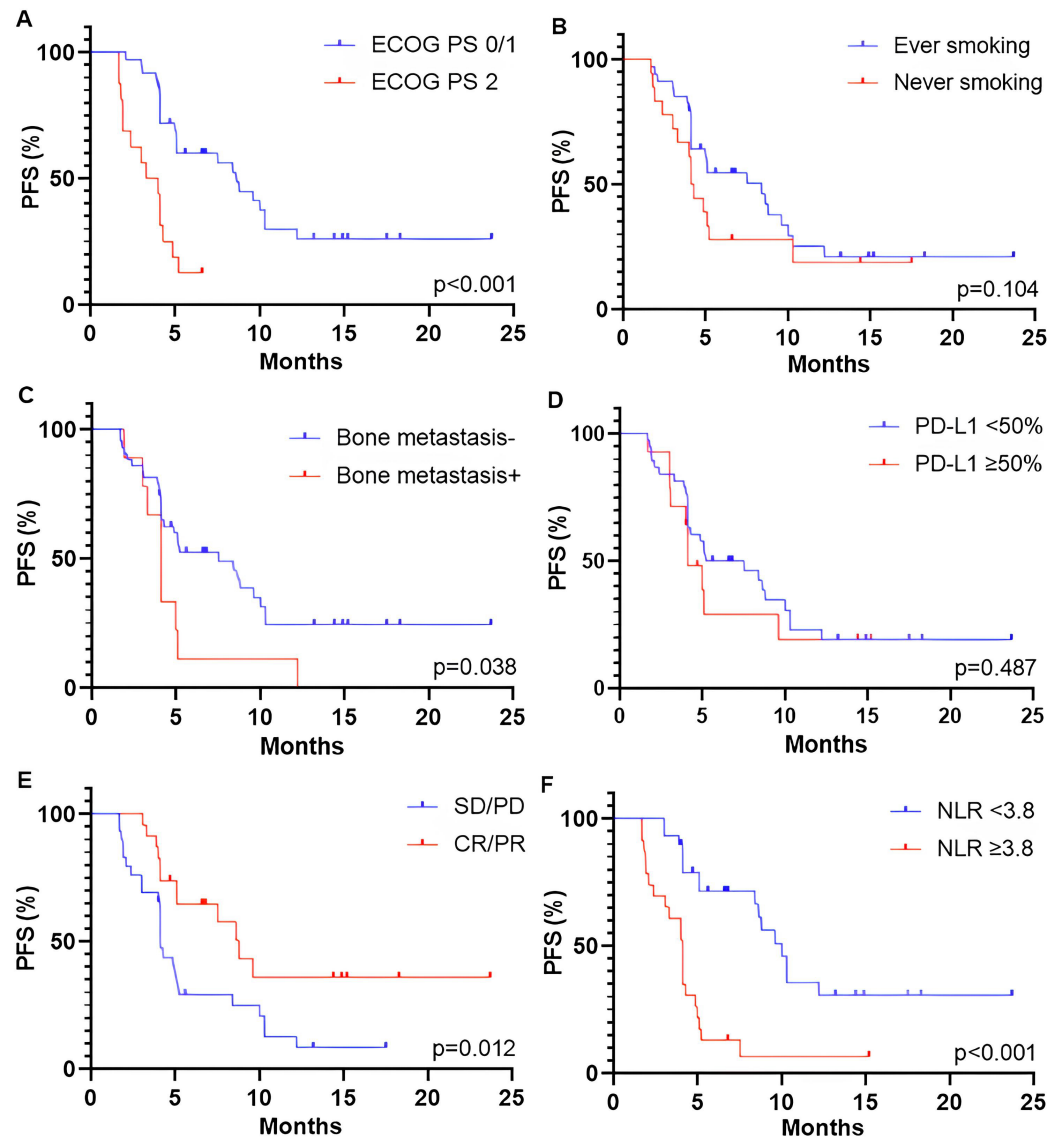
Kaplan-Meier curves for PFS of patients who received ICI rechallenge based on different parameters. (A) ECOG PS at the start of ICI rechallenge; (B) smoking status; (C) PD-L1 expression; (D) bone metastasis; (E) best overall response to initial ICI therapy; and (F) NLR at the baseline of ICI rechallenge. PFS: progression-free survival; ECOG PS: Eastern Cooperative Oncology Group Performance Status; PD-L1: programmed cell death-ligand 1; SD: stable disease; PD: progressive disease; CR: complete response; PR: partial response; NLR: neutrophil-to-lymphocyte ratio; ICI: immune checkpoint inhibitor

**Figure 3:**
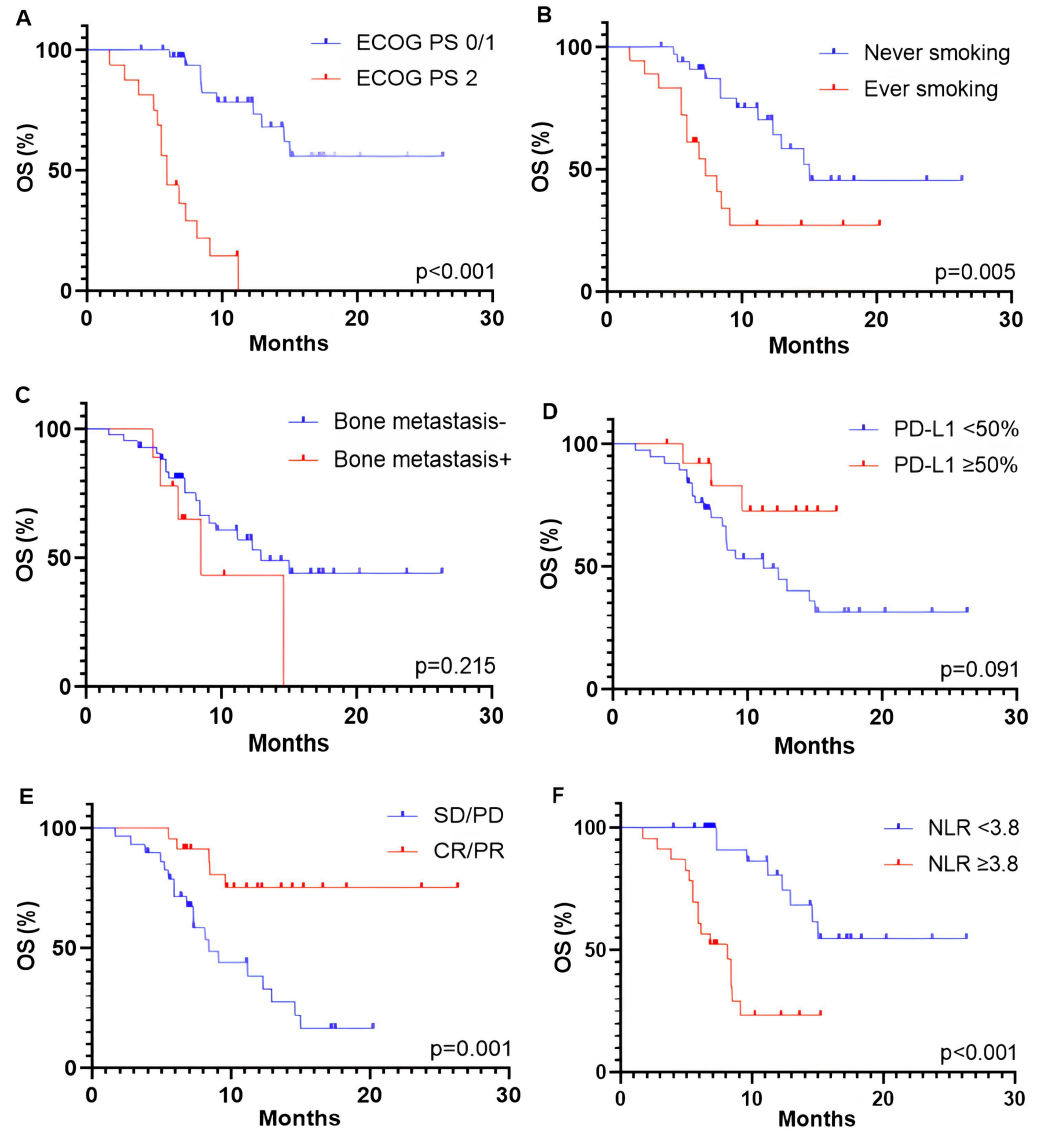
Kaplan-Meier curves for OS of patients receiving ICI rechallenge based on different characteristics, including (A) ECOG PS at the start of ICI rechallenge, (B) smoking status, (C) PD-L1 expression, (D) bone metastasis, (E) best overall response to initial ICI therapy, and (F) NLR at the baseline of ICI rechallenge. OS: overall survival; ECOG PS: Eastern Cooperative Oncology Group Performance Status; PD-L1: programmed cell death-ligand 1; SD: stable disease; PD: progressive disease; CR: complete response; PR: partial response; NLR: neutrophil-to-lymphocyte ratio; ICI: immune checkpoint inhibitor

The median PFS and OS were 5.1 months (95% CI, 4.1–8.8 months) and 12.9 months (95% CI, 8.4–15.0 months), respectively. The association between survival outcomes and clinical and pathological characteristics was shown in Figure 2 (for PFS) and Figure 3 (for OS). Patients with a good performance status (ECOG PS 0/1) at the start of ICI rechallenge therapy exhibited longer PFS and OS compared to patients with ECOG PS 2 (PFS: 8.6 months vs. 3.7 months, *P* < 0.001, Figure 2A; OS: not reached vs. 5.9 months, *P* < 0.001, Figure 3A). Presence of smoking history was associated with longer OS (15.0 months vs. 7.3 months, *P* = 0.005; Figure 3B), but this relationship was not statistically significant for PFS (*P* = 0.104; Figure 2B). Presence of bone metastasis at the start of ICI rechallenge was statistically significantly associated with a shorter PFS (4.1 months vs. 7.5 months, *P* = 0.038; Figure 2С), but not with OS (*P* = 0.215; Figure 3C).

The optimal threshold level of PD-L1 expression was defined as ≥ 50%. However, only a trend towards longer OS was observed in patients with PD-L1 ≥ 50% (*P* = 0.091; Figure 3D), with no association found for PFS (*P* = 0.487; Figure 2D). Patients who had an objective response (partial and CR) to initial ICI therapy showed longer PFS and OS compared to those with stable and PD (PFS: 8.8 months vs. 4.1 months, *P* = 0.012, Figure 2E; OS: not reached vs. 8.4 months, *P* = 0.001, Figure 3E). A baseline NLR below the cut-off value of 3.8 before starting ICI rechallenge is associated with longer PFS (10.0 months vs. 4.1 months, *P* < 0.001; Figure 2F) and OS (not reached vs. 8.1 months, *P* < 0.001; Figure 3F).

The univariate analysis demonstrated a significant association between shorter PFS and several parameters such as ECOG PS 2 [hazard ratio (HR), 5.31; 95% CI, 2.46–11.45; *P* < 0.001], presence of bone metastasis at the start of ICI rechallenge (HR, 2.33; 95% CI, 1.02–4.81; *P* = 0.030), absence of objective response (partial and CR) during initial ICI therapy (HR, 0.41; 95% CI, 0.19–0.89; *P* = 0.026), and baseline NLR ≥ 3.8 (HR, 6.76; 95% CI, 3.17–15.48; *P* < 0.001) (Table 2). In multivariate analysis, only ECOG PS 2 (HR, 3.41; 95% CI, 1.28–9.05; *P* = 0.013), absence of objective response during initial ICI therapy (HR, 0.34; 95% CI, 0.16–0.78; *P* = 0.014), and NLR ≥ 3.8 at the baseline of ICI rechallenge (HR, 5.89; 95% CI, 2.29–16.20; *P* = 0.001) were found to be associated with shorter PFS (Table 2).

**Table 2:** Univariate and multivariate analyses for PFS using Cox proportional hazards regression model in ICI rechallenge cohort

**Characteristics**	**Univariate analysis**		**Multivariate analysis**	
	**HR (95% CI)**	***P***	**HR (95% CI)**	***P***
Age	0.98 (0.96–1.06)	0.181	-	-
Gender (male vs. female)	0.65 (0.33–1.43)	0.254	-	-
BMI	0.96 (0.89–1.03)	0.265	-	-
ECOG PS at ICI rechallenge (2 vs. 0/1)	5.31 (2.46–11.45)	< 0.001	3.41 (1.28–9.05)	0.013
Smoking (never vs. ever)	1.79 (0.91–3.44)	0.084	-	-
Histology (squamous vs. non-squamous)	0.62 (0.32–1.22)	0.169	-	-
PD-L1 expression (< 50% vs. ≥ 50%)	1.30 (0.59–2.62)	0.481	-	-
Liver metastasis at ICI rechallenge (yes vs. no)	1.03 (0.47–2.06)	0.941	-	-
Brain metastasis at ICI rechallenge (yes vs. no)	2.06 (0.60–5.38)	0.182	-	-
Bone metastasis at ICI rechallenge (yes vs. no)	2.33 (1.02–4.81)	0.030	1.59 (0.58–4.19)	0.356
Metastatic sites at ICI rechallenge (< 2 vs. ≥ 2)	1.83 (0.74–3.94)	0.152	-	-
Line of initial ICI therapy (1 vs. 2)	0.64 (0.22–1.52)	0.362	-	-
Initial ICI regimen (monotherapy vs. combination chemotherapy)	0.83 (0.37–1.71)	0.636	-	-
Best overall response to initial ICI therapy (CR/PR vs. SD/PD)	0.41 (0.19–0.89)	0.026	0.34 (0.16–0.78)	0.014
Duration of initial ICI therapy (≥ 1 year vs. < 1 year)	0.56 (0.26–1.11)	0.107	-	-
irAEs at initial ICI therapy (yes vs. no)	1.11 (0.57–2.12)	0.758	-	-
Treatment between two lines of ICI (yes vs. no)	1.58 (0.83–3.09)	0.169	-	-
ICI rechallenge regimen (monotherapy vs. combination chemotherapy)	0.77 (0.34–2.04)	0.552	-	-
Type of ICIs in rechallenge settings [anti-PD-L1 (atezolizumab) vs. anti-PD-1 (pembrolizumab/nivolumab)]	1.13 (0.55–2.40)	0.741	-	-
Rechallenge with the same ICI (no vs. yes)	0.99 (0.52–1.94)	0.982	-	-
Line of ICI rechallenge therapy (< 4 vs. ≥ 4)	0.58 (0.28–1.31)	0.158	-	-
Baseline NLR at ICI rechallenge (≥ 3.8 vs. < 3.8)	6.76 (3.17–15.48)	< 0.001	5.89 (2.25–16.20)	0.001
irAEs at ICI rechallenge (yes vs. no)	1.08 (0.52–2.11)	0.830	-	-

BMI: body mass index; ECOG PS: Eastern Cooperative Oncology Group Performance Status; NLR: neutrophil-to-lymphocyte ratio; irAEs: immune-related adverse events; HR: hazard ratio; SD: stable disease; PD: progressive disease; CR: complete response; PR: partial response; PFS: progression-free survival; PD-L1: programmed cell death-ligand 1; ICI: immune checkpoint inhibitor; PD-1: programmed cell death protein 1

Univariate analysis of the patients’ data showed that ECOG PS 2 (HR, 6.84; 95% CI, 2.87–13.68; *P* < 0.001), no history of smoking (HR, 3.28; 95% CI, 1.45–7.56; *P* = 0.004), absence of objective response during initial ICI therapy (HR, 0.06; 95% CI, 0.01–0.22; *P* = 0.002), and baseline NLR ≥ 3.8 at ICI rechallenge (HR, 6.59; 95% CI, 2.71–12.92; *P* < 0.001) were associated with shorter OS (Table 3). The multivariate analysis for OS revealed that only ECOG PS 2 (HR, 4.51; 95% CI, 1.30–15.89; *P* = 0.037), absence of objective response to initial ICI therapy (HR, 0.19; 95% CI, 0.04–0.80; *P* = 0.028), and baseline NLR ≥ 3.8 (HR, 6.80; 95% CI, 1.95–13.84; *P* = 0.003) were negative predictive factors (Table 3).

**Table 3:** Univariate and multivariate analyses for OS using Cox proportional hazards regression model in ICI rechallenge cohort

**Characteristics**	**Univariate analysis**		**Multivariate analysis**	
	**HR (95% CI)**	***P***	**HR (95% CI)**	***P***
Age	0.98 (0.95–1.02)	0.264	-	-
Gender (male vs. female)	0.77 (0.32–2.13)	0.581	-	-
BMI	0.96 (0.87–1.04)	0.350	-	-
ECOG PS at ICI rechallenge (2 vs. 0/1)	6.84 (2.87–13.68)	< 0.001	4.51 (1.30–15.89)	0.037
Smoking (never vs. ever)	3.28 (1.45–7.56)	0.004	1.28 (0.37–4.28)	0.686
Histology (squamous vs. non-squamous)	1.84 (0.73–4.37)	0.234	-	-
PD-L1 expression (< 50% vs. ≥ 50%)	2.72 (0.94–11.52)	0.106	-	-
Liver metastasis at ICI rechallenge (yes vs. no)	1.66 (0.67–3.80)	0.241	-	-
Brain metastasis at ICI rechallenge (yes vs. no)	1.44 (0.34–4.31)	0.560	-	-
Bone metastasis at ICI rechallenge (yes vs. no)	2.39 (0.85–5.84)	0.071	-	-
Metastatic sites at ICI rechallenge (< 2 vs. ≥ 2)	2.07 (0.68–5.23)	0.151	-	-
Line of initial ICI therapy (1 vs. 2)	0.43 (0.07–1.45)	0.249	-	-
Initial ICI regimen (monotherapy vs. combination chemotherapy)	0.67 (0.22–1.68)	0.430	-	-
Best overall response to initial ICI therapy (CR/PR vs. SD/PD)	0.06 (0.01–0.22)	0.002	0.19 (0.04–0.80)	0.028
Duration of initial ICI therapy (≥ 1 year vs. < 1 year)	0.56 (0.22–1.29)	0.196	-	-
irAEs at initial ICI therapy (yes vs. no)	0.60 (0.24–1.37)	0.243	-	-
Treatment between two lines of ICI (yes vs. no)	1.28 (0.57–2.98)	0.553	-	-
ICI rechallenge regimen (monotherapy vs. combination chemotherapy)	0.66 (0.15–1.92)	0.500	-	-
Type of ICIs in rechallenge settings [anti-PD-L1 (atezolizumab) vs. anti-PD-1 (pembrolizumab/nivolumab)]	1.13 (0.44–3.00)	0.794	-	-
Rechallenge with the same ICI (no vs. yes)	0.83 (0.36–1.87)	0.663	-	-
Line of ICI rechallenge therapy (< 4 vs. ≥ 4)	0.48 (0.19–1.48)	0.158	-	-
Baseline NLR at ICI rechallenge (≥ 3.8 vs. < 3.8)	6.59 (2.71–12.92)	< 0.001	6.80 (1.95–13.84)	0.003
irAEs at ICI rechallenge (yes vs. no)	0.69 (0.25–1.66)	0.436	-	-

BMI: body mass index; ECOG PS: Eastern Cooperative Oncology Group Performance Status; NLR: neutrophil-to-lymphocyte ratio; irAEs: immune-related adverse events; HR: hazard ratio; OS: overall survival; SD: stable disease; PD: progressive disease; CR: complete response; PR: partial response; PD-L1: programmed cell death-ligand 1; ICI: immune checkpoint inhibitor; PD-1: programmed cell death protein 1

### Predictive score of ICI rechallenge survival outcomes

**Figure 4:**
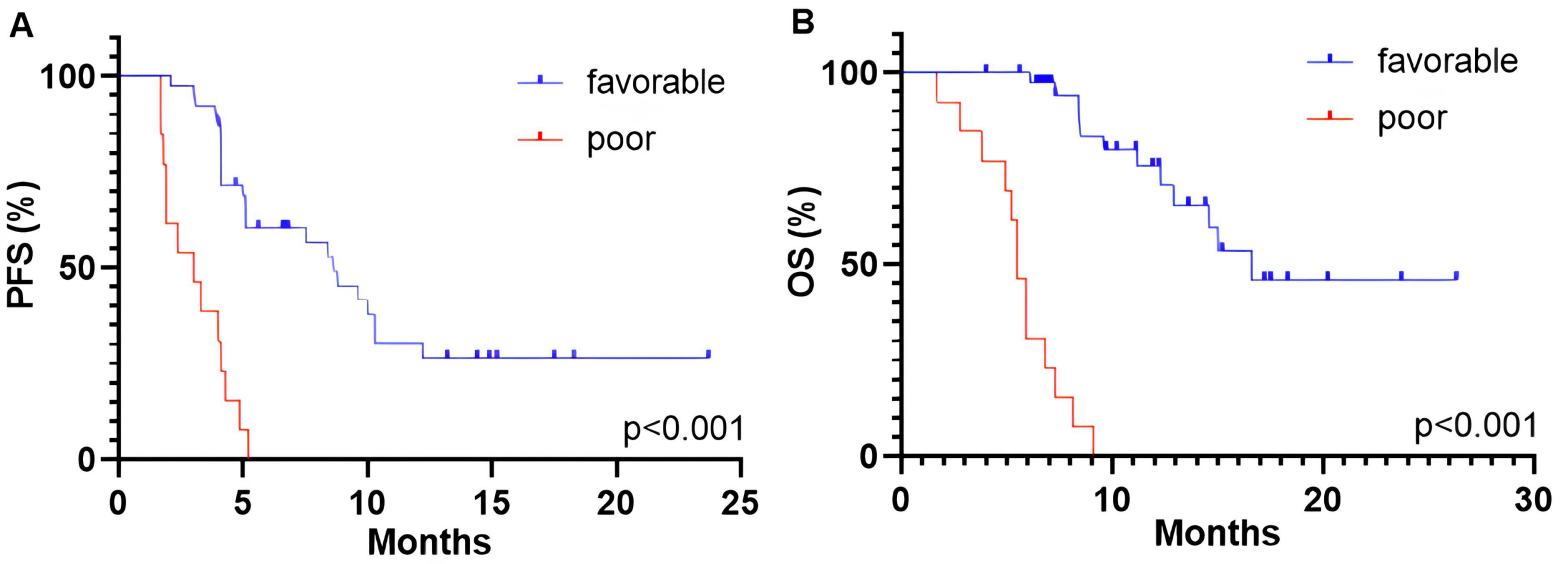
Kaplan-Meier curves for PFS (A) and OS (B) dividing patients into two predictive groups based on the NEO score: favorable and poor. PFS: progression-free survival; OS: overall survival

The multivariate analysis for both PFS and OS showed that independent positive predictive factors were baseline NLR < 3.8 at ICI rechallenge, ECOG PS 0-1 and objective response to initial ICI therapy. The risk model that included these three parameters was referred to as the NEO score, which is an acronym for the first letters of the markers. The survival outcomes of patients were stratified into two predictive groups based on the NEO score: favorable (presence of at least one marker—NLR < 3.8, ECOG PS 0-1, objective response) and poor (absence of all three markers). Patients with a favorable NEO score (*n* = 13, 25%) were associated with longer PFS than those with a poor NEO score (*n* = 39, 75%) (8.6 months vs. 3.0 months, *P* < 0.001; Figure 4A). The median OS was 16.6 months for the favorable group and 5.5 months for the poor predictive group (*P* < 0.001; Figure 4B). There was no association observed between the NEO score groups and ORR (*P* > 0.05).

In univariate analysis, patients with a poor NEO score showed shorter PFS (HR, 12.06; 95% CI, 4.28–36.48; *P* < 0.001) and OS (HR, 6.82; 95% CI, 3.10–14.86; *P* < 0.001) compared to those in a favorable predictive group. This association also remained significant in the multivariate analysis (Table 4).

**Table 4:** Multivariate analysis for PFS and OS based on the NEO score

**NEO score group**	**PFS**		**OS**	
	**HR (95% CI)**	***P***	**HR (95% CI)**	***P***
Favorable	1 [Reference]		1 [Reference]	
Poor	7.30 (2.62–20.84)	< 0.001	15.34 (4.16–36.90)	< 0.001

HR: hazard ratio; PFS: progression-free survival; OS: overall survival

### Efficacy of standard chemotherapy and comparison with ICI rechallenge

**Figure 5:**
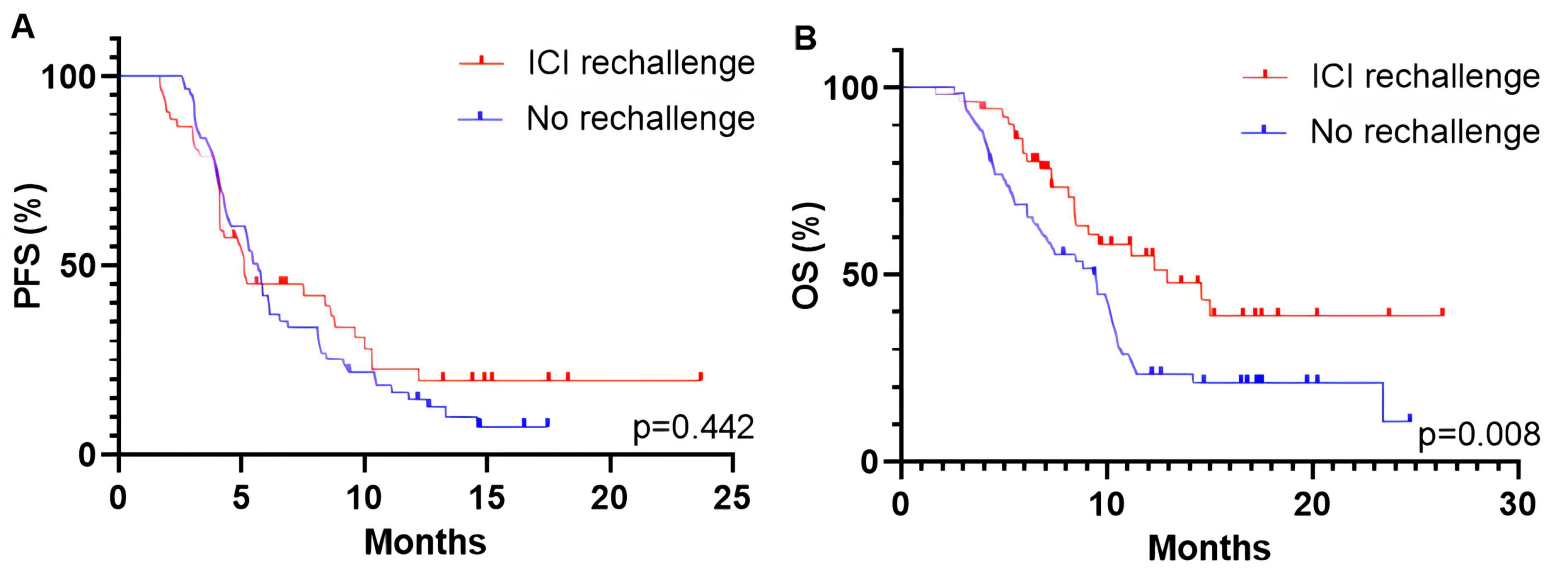
Kaplan-Meier curves for PFS (A) and OS (B) of patients in ICI rechallenge and no rechallenge cohorts. PFS: progression-free survival; OS: overall survival; ICI: immune checkpoint inhibitor

The ORR in patients receiving subsequent line chemotherapy was 9/61 (14.8%). The median PFS and OS were 5.7 months and 9.4 months, respectively. There were no statistically significant differences in ORR and PFS between the rechallenge and no rechallenge cohorts (ORR: 5.8% vs. 14.8%, *P* = 0.140; PFS: 5.1 months vs. 5.7 months, *P* = 0.442, Figure 5A). Patients receiving ICI rechallenge therapy exhibited longer OS compared to those who did not undergo ICI retreatment (12.9 months vs. 9.6 months, *P* = 0.008; Figure 5B).

There was no significant association between the clinical, morphological, and treatment parameters and ORR (*P* > 0.05). In univariate analysis, only ECOG PS 2 at the start of chemotherapy was associated with a shorter OS (HR, 2.33; 95% CI, 1.18–4.38; *P* = 0.011); however, there was no significant tendency for a shorter PFS (Table 5). Multivariate analysis for OS also showed that only ECOG PS 2 (HR, 2.25; 95% CI, 1.07–4.55; *P* = 0.027) was a negative prognostic factor.

**Table 5:** Univariate analysis for PFS and OS using Cox proportional hazards regression model in chemotherapy cohort

**Characteristics**	**PFS**		**OS**	
	**HR (95% CI)**	***P***	**HR (95% CI)**	***P***
Age	1.03 (0.93–1.15)	0.594	0.99 (0.97–1.02)	0.522
Gender (male vs. female)	0.31 (0.02–2.31)	0.316	0.75 (0.40–1.51)	0.391
BMI	0.93 (0.70–1.21)	0.596	0.97 (0.90–1.04)	0.404
ECOG PS (2 vs. 0/1)	1.67 (0.87–3.02)	0.102	2.33 (1.18–4.38)	0.011
Smoking (never vs. ever)	1.42 (0.83–2.45)	0.198	1.26 (0.70–2.26)	0.432
Histology (squamous vs. non-squamous)	1.11 (0.64–1.96)	0.721	1.23 (0.67–2.20)	0.489
PD-L1 expression (< 50% vs. ≥ 50%)	1.33 (0.66–2.46)	0.393	1.50 (0.71–2.93)	0.259
Brain metastasis at subsequent line chemotherapy (yes vs. no)	1.59 (0.48–3.96)	0.379	2.29 (0.66–5.76)	0.134
Bone metastasis at subsequent line chemotherapy (yes vs. no)	1.09 (0.45–2.27)	0.837	1.08 (0.47–3.13)	0.876
Metastatic sites at subsequent line chemotherapy (< 2 vs. ≥ 2)	1.00 (0.58–1.71)	0.989	0.87 (0.48–1.57)	0.643
Line of initial ICI therapy (1 vs. 2)	0.78 (0.30–1.70)	0.568	0.65 (0.20–1.62)	0.416
Initial ICI regimen (monotherapy vs. combination chemotherapy)	1.22 (0.67–2.38)	0.542	1.34 (0.68–2.97)	0.429
Best overall response to initial ICI therapy (CR/PR vs. SD/PD)	0.92 (0.49–1.69)	0.790	0.83 (0.43–1.54)	0.574
Duration of initial ICI therapy (≥ 1 year vs. < 1 year)	0.90 (0.51–1.56)	0.721	0.62 (0.33–1.11)	0.115
irAEs at initial ICI therapy (yes vs. no)	0.68 (0.39–1.18)	0.169	0.90 (0.51–1.62)	0.729
Subsequent line treatment regimen (monotherapy vs. combination chemotherapy)	1.61 (0.88–3.14)	0.140	1.43 (0.77–2.82)	0.277
Baseline NLR at subsequent line therapy (≥ 3.8 vs. < 3.8)	1.22 (0.71–2.09)	0.468	1.58 (0.89–2.83)	0.121

PFS: progression-free survival; OS: overall survival; BMI: body mass index; ECOG PS: Eastern Cooperative Oncology Group Performance Status; NLR: neutrophil-to-lymphocyte ratio; irAEs: immune-related adverse events; HR: hazard ratio; PD-L1: programmed cell death-ligand 1; SD: stable disease; PD: progressive disease; CR: complete response; PR: partial response; ICI: immune checkpoint inhibitor

The predictive significance of the NEO score was also evaluated in the cohort that did not undergo rechallenge. Based on the score, 11 patients (18.0%) were stratified into the poor prognostic group, while 50 (82.0%) were stratified into the favorable group. No differences were showed between the NEO score groups and ORR (*P* = 0.367). In univariate analysis, no significant association was observed between prognostic groups and survival outcomes (PFS: HR, 1.27; 95% CI, 0.60–2.44; *P* = 0.491; OS: HR, 1.67; 95% CI, 0.78–3.25; *P* = 0.193).

## Discussion

In the present study, we identified several clinical parameters that predict the efficacy of ICI rechallenge therapy. These markers include smoking history, ECOG PS and bone metastasis at ICI rechallenge, best overall response to initial ICI therapy, and baseline NLR at the start of subsequent therapy. The combination of baseline NLR, ECOG PS, and best overall response to initial therapy accurately stratified patients who would benefit from ICI rechallenge. Additionally, the combination of these markers was found to be predictive for ICI rechallenge, rather than prognostic, as it did not show a relationship with survival outcomes in patients who did not undergo ICI rechallenge. The ICI rechallenge also demonstrated an OS advantage over standard chemotherapy in patients who had initially received ICI therapy regardless of stratifying patients by predictive markers. The results of a recent meta-analysis provided some support for our findings [10]. In the study by Feng et al. [10], the median OS for ICI rechallenge was 13.1 months. It is important to note that our study was retrospective, meaning that ICI rechallenge was prescribed primarily to patients who had experienced substantial clinical benefit from the initial ICI therapy. In order to properly compare two cohorts, patients for the no rechallenge cohort were selected who had previously received ICI and a platinum-containing doublet for metastatic disease, and who had a similar response pattern to initial therapy. Additionally, patients in both cohorts had comparable functional status, tumor burden and proportion of positive PD-L1 expression.

In prospective studies, high levels of tumor PD-L1 expression was reported to be associated with the response to initial ICI therapy in metastatic NSCLC [13, 23]. A retrospective study of 12 patients who underwent ICI rechallenge treatment showed that all patients with PR and SD had very high PD-L1 levels of ≥ 80% [24]. A post-hoc analysis of three phase III trials revealed that patients who had PD-L1 positive NSCLC had high disease control rates (complete, PR and SD) during ICI rechallenge therapy, ranging from 75.8% to 83.3% [25–27]. However, in these studies, all patients were long-term survivors and received ICI rechallenge after completing of 2 years of pembrolizumab therapy and experiencing subsequent disease progression [25–27]. The current study demonstrated that patients with PD-L1 ≥ 50% only tended to have longer OS during ICI rechallenge therapy. Additionally, several retrospective studies showed a lack of significant association between the PD-L1 expression level and the efficacy of the retreatment option [15, 28, 29]. The main limitation of using PD-L1 expression as a predictive marker, both in our work and in published studies, is the assessment of the expression before the start of initial ICI therapy. This approach does not allow us to assess the change in PD-L1 expression under the exposure of the first therapy [16]. Therefore, the predictive value of this marker may be enhanced if it is determined before the start of ICI rechallenge [16].

Smoking status is one of the predictive clinical markers of the ICI efficacy [30]. Smoking history is associated with a higher mutational burden, resulting in increased tumor immunogenicity, an anti-tumor immune microenvironment profile, and an upregulation of PD-L1 expression [30]. A recent meta-analysis showed that smokers who received initial ICI therapy experienced a benefit in OS [31]. However, this relationship was not observed in studies investigating predictive markers of ICI rechallenge efficacy in NSCLC patients [10, 28]. In our study, smokers had an advantage in OS, but this association did not persist in multivariate analysis.

Another clinical marker that may be used as predictive is the presence of bone metastasis. This factor serves as a negative predictor of response to immunotherapy due to the presence of an immunosuppressive tumor and bone microenvironment in these patients [32]. The bone metastasis in the present study were only associated with a shorter PFS, which is consisted with the study conducted by Gobbini et al. [33].

The general status that is determined stratifying by ECOG PS was a prognostic factor of survival outcomes regardless of treatment in NSCLC [34]. The present study showed a relationship between ECOG PS 2 and a shorter OS in patients who received chemotherapy in the no rechallenge cohort. Additionally, poor performance status was associated with reduced efficacy of ICI therapy due to the deteriorated immune status of the patient, especially the impaired effector T cell response [35, 36]. Similar to the previous studies [15, 29, 33], patients with poor performance status before the start of ICI rechallenge therapy had shorter PFS and OS.

Patients who achieved a partial or CR to initial ICI therapy experienced a survival benefit from ICI rechallenge therapy. Feng et al. [28] also demonstrated that an objective response to initial ICI treatment was an independent predictive marker of longer PFS during ICI rechallenge therapy. The rationale for these findings is that some of sensitive clones may persist after initial therapy and regrow during the immunotherapy-free interval or exposure to other treatment options [37, 38].

NLR is a reliable and cost-effective marker of systemic inflammation in cancer [39]. The interaction between neutrophils and lymphocytes reflects the equilibrium between cancer-related inflammation and anti-tumor activity [40]. NLR is associated with a poor prognosis in NSCLC regardless of the treatment option [39]. Additionally, a high NLR predicts negative survival outcomes for chemotherapy [39], however, this association was not observed in ICI-pretreated NSCLC patients who received subsequent-line chemotherapy. NLR is a well-established predictive marker of resistance to initial ICI treatment in metastatic NSCLC [40]. A high NLR was independently associated with shorter PFS and OS only in patients who received ICI rechallenge, which is consisted with the previous studies [15, 41].

Predictive scores, which incorporate a range of different indicators, have the potential to improve the accuracy of identifying responders to ICI treatment, rather than relying solely depending on a single predictive marker. Several scales have been proposed to predict the efficacy of initial ICI therapy in metastatic NSCLC [42–45]. However, prior to the current study, none of the studies on ICI rechallenge had suggested a predictive nomogram. Our predictive score, named the NEO score, includes individual parameters that are independent predictors of survival benefit. Patients in the favorable group of the NEO score, who had at least one of the markers NLR < 3.8, ECOG PS 0-1, or objective response, experienced significantly longer PFS and OS compared to patients in the poor group who did not have any of these parameters. Furthermore, there was no association found between the NEO score and survival outcomes for chemotherapy in patients who did not undergo ICI retreatment. This emphasizes the predictive value of the score in relation to the ICI rechallenge. The investigation of a predictive score had some limitations: a retrospective nature of the study and a lack of evaluation of PD-L1 expression before the start of ICI rechallenge. However, despite these limitations the NEO score represents an easy-to-access and worldwide routinely available predictive tool that could assist in decision-making for ICI rechallenge therapy of metastatic NSCLC.

In conclusion, ICI rechallenge demonstrated a survival benefit in ICI-pretreated NSCLC, particularly in patients with NLR < 3.8, ECOG PS 0-1, and objective response. Furthermore, the NEO score, which is based on these markers, was able to accurately predict PFS and OS. However, additional prospective studies are warranted to confirm these findings.

## Abbreviations

BMI: body mass index
CR: complete response
ECOG PS: Eastern Cooperative Oncology Group Performance Status
HR: hazard ratio
ICI: immune checkpoint inhibitor
IHC: immunohistochemistry
irAEs: immune-related adverse events
NLR: neutrophil-to-lymphocyte ratio
NSCLC: non-small cell lung cancer
ORR: objective response rate
OS: overall survival
PD: progressive disease
PD-1: programmed cell death protein 1
PD-L1: programmed cell death-ligand 1
PFS: progression-free survival
PR: partial response
SD: stable disease

## Declarations

### Availability of data and materials

The raw data supporting the conclusions of this manuscript will be made available by the corresponding author (a.musaelyan8@gmail.com), without undue reservation, to any qualified researcher.

### Copyright

© The Author(s) 2024.
